# A Meta-Analysis: Identification of Common Mir-145 Target Genes that have Similar Behavior in Different GEO Datasets

**DOI:** 10.1371/journal.pone.0161491

**Published:** 2016-09-21

**Authors:** Elnaz Pashaei, Esra Guzel, Mete Emir Ozgurses, Goksun Demirel, Nizamettin Aydin, Mustafa Ozen

**Affiliations:** 1 Department of Computer Engineering, Yildiz Technical University, Istanbul, Turkey; 2 Biruni University, Department of Molecular Biology and Genetics, Topkapi, Istanbul, Turkey; 3 Department of Medical Genetics, Istanbul University Cerrahpasa Medical School, Istanbul, Turkey; 4 Department of Pathology & Immunology Baylor College of Medicine, Houston, Texas, 77030, United States of America; University of São Paulo, BRAZIL

## Abstract

**Background:**

MicroRNAs, which are small regulatory RNAs, post-transcriptionally regulate gene expression by binding 3'-UTR of their mRNA targets. Their deregulation has been shown to cause increased proliferation, migration, invasion, and apoptosis. miR-145, an important tumor supressor microRNA, has shown to be downregulated in many cancer types and has crucial roles in tumor initiation, progression, metastasis, invasion, recurrence, and chemo-radioresistance. Our aim is to investigate potential common target genes of miR-145, and to help understanding the underlying molecular pathways of tumor pathogenesis in association with those common target genes.

**Methods:**

Eight published microarray datasets, where targets of mir-145 were investigated in cell lines upon mir-145 over expression, were included into this study for meta-analysis. Inter group variabilities were assessed by box-plot analysis. Microarray datasets were analyzed using GEOquery package in Bioconducter 3.2 with R version 3.2.2 and two-way Hierarchical Clustering was used for gene expression data analysis.

**Results:**

Meta-analysis of different GEO datasets showed that UNG, FUCA2, DERA, GMFB, TF, and SNX2 were commonly downregulated genes, whereas MYL9 and TAGLN were found to be commonly upregulated upon mir-145 over expression in prostate, breast, esophageal, bladder cancer, and head and neck squamous cell carcinoma. Biological process, molecular function, and pathway analysis of these potential targets of mir-145 through functional enrichments in PPI network demonstrated that those genes are significantly involved in telomere maintenance, DNA binding and repair mechanisms.

**Conclusion:**

As a conclusion, our results indicated that mir-145, through targeting its common potential targets, may significantly contribute to tumor pathogenesis in distinct cancer types and might serve as an important target for cancer therapy.

## Introduction

MicroRNAs (miRNAs) are 18–22 nucleotide long, non-coding RNAs, which are transcribed by RNA polymerase II. They regulate their target genes expression at the post-transcriptional level through binding to the 3’-untranslated regions (3’ UTR) of specific mRNAs and either cause mRNA degradation or translational inhibition [1, 2]

MiRNAs are implicated in several central biological processes such as cell development, proliferation, differentiation, and apoptosis. They also have been shown to possess crucial roles in cancer initiation, progression, and metastasis [3, 4].

The involvement of miRNAs in cancer pathogenesis is well established, as they can behave as oncogenes or tumor suppressor genes depending on the cellular functions of their targets. As tumor suppressors, miRNAs are downregulated in cancer tissues and repress their oncogenic targets. On the other hand, some miRNAs are upregulated in tumor samples and trigger cancer growth. Additionally, both tumor suppressor and oncogenic miRNAs can induce multiple cancer traits by targeting different cellular pathways [5].

Various pathways can be affected by deregulation of miRNAs, since a single miRNA can target hundreds of mRNAs in the same or different biological pathways, and they can regulate the expressions of human genes up to %60 [6].

DNA Microarray is one such technology, which provides profiling of thousands of genes expressions at the same time. Microarrays are effective tools in cancer research, where they can be used to predict tumor development and progression, to evaluate drug response, and to find out biomarkers [7].

miR-145 is a well-studied miRNA, which is located at 5p32 chromosomal region and its expression is controlled by p53 and some other transcriptional factors like RREB1, FoxO, and C/EBP- β [8].

Mir-145 acts as a tumor suppressor and has been shown to be downregulated in several cancer types including prostate [9, 10], head and neck [11], pancreatic ductal adenocarcinoma [12], lung [13], breast [14], colorectal [15, 16], bladder [17], and gastric cancer [18].

It promotes apoptosis in the growing cells by silencing MYC (MYC-c), PPP3CA, EGFR, NUDT1, TNSF10, SWAP70, DEFA, CBFB, CLINT1, and RTKN [19].

miR-145 has been found to be associated with tumorigenesis via suppressing the expression of several genes such as Insulin-like growth factor 1 in colorectal cancer [20], c-Myc and Cyclin-dependent kinase 6 (Cdk6) in oral squamous cell cancer [21], ER-α in breast cancer, SOX2 in larynx and prostate cancer, and several other genes in distinct cancer types [22]. However, to the best of our knowledge there is no a meta-analysis study investigating mir-145 targets and this is the first study, which combines and correlates miR-145 and mRNA microarray data in the literature.

In this study, we aimed to show potential common target genes of miR-145 in several cancer types including prostate, breast, esophageal, bladder, head, and neck squamous cell carcinoma cancer, using GEO database and to unravel the underlying molecular pathways associated with mir-145 in tumor pathogenesis.

## Material and Methods

### Literature search

**Fig 1:**
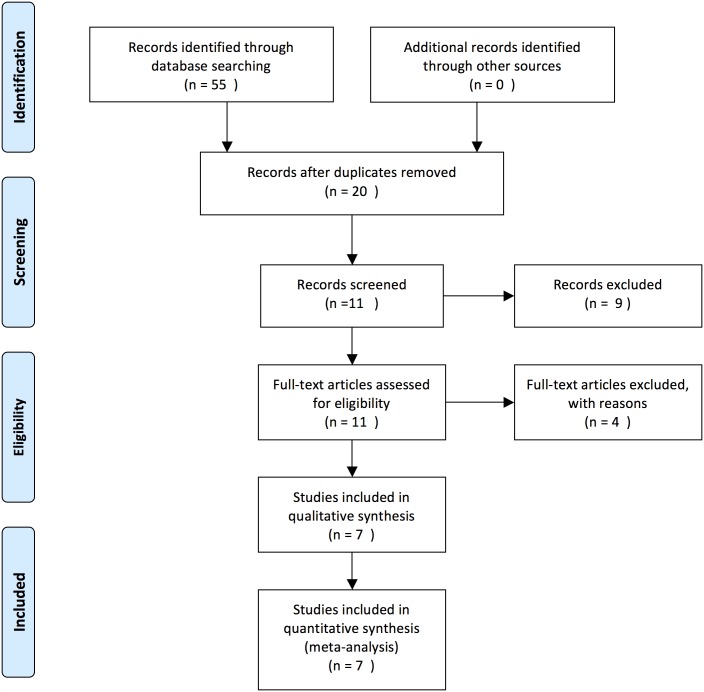
Flow chart of study selection in the meta-analysis.

A systematic review of the microarray literature from GEO database was documented to identify studies, where expression profiling was performed for miR-145 over-expressing cancer cell lines, published up to Jun 15, 2014. Medical subjective heading (MeSH) was “miR-145 in human cancer”. A total of 55 studies were identified through GEO database searching. Of these, 20 studies were retained after rejecting replications. A total of 9 articles were excluded according to the title of samples (GSMs). The reason for the exclusion was the following: the title of samples no association with miR-145. The full-text articles were evaluated for the remaining 11 studies, and 7 were recruited in the final meta-analysis. The other 4 investigations were excluded for these reasons: the median-centered across samples is not zero (not suitable for comparison) and data sets are containing null values. Remained 7 Microarray datasets were obtained from National Centers for Biotechnology Information (NCBI) Gene Expression Omnibus (GEO) database (http://www.ncbi.nlm.nih.gov/geo/). The entire study selection process for meta-analysis is shown in Fig 1.

All GSE Series Matrix files, platform sets and annotations files were downloaded and parsed by GEOquery package in Bioconducter 3.2 with R version 3.2.2. Box-plots were drawn for each selected GSE microarray data for visual comparison of inter-group variability in a statistical population. These box plots were used to display the statistical distribution of different data values of mir-145 GSMs.

### Data Preparation and Statistical Analysis

**Fig 2:**
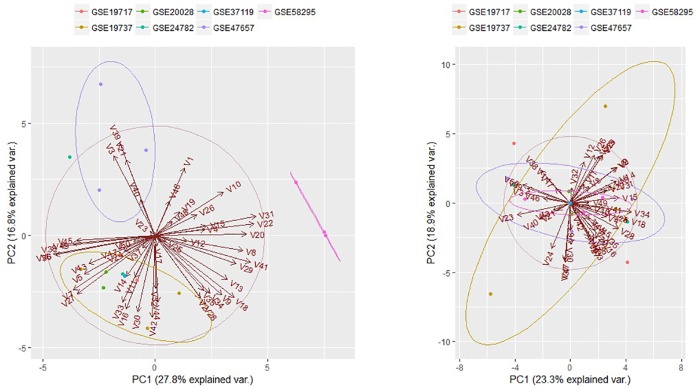
Principal component analysis (PCA) plot for combined dataset before (left panel) and after (right panel) removing batch effect.

Before being able to do statistical analysis, we need to be prepared to meet the following requirements: raw data must be log2- scaled and all datasets must exhibit the same data precision. In our dataset except GSE58295 all of GSEs were in the form of (log)_10_ (ratio), therefore, we converted them to (log)_2_ (ratio) format. All GSEs have 16 bit precision. Since the comparable data are provided, the analysis should be limited to genes that are expressed in all data sets. In 16 GSMs (samples) 17085 common genes were founded. Then, we checked for the cross-platform bias (batch effect) by computing and plotting the Principal Components Analysis (PCA) for combined dataset. PCA plot (Fig 2, left panel) shows that sample of the each technology (GSE) clusters together. This means that we have batch effect in our data. We have removed batch effect by “removeBatchEffect” function in limma package. The right panel of Fig 2 shows PCA plot after removing the batch effect. Note that the expression values of combined data will change after removing batch effect. In the next step we try to design and make proper contrast matrix again by limma package. We have five groups (each disease) with eight replicates in group1, one replicate in group2, three replicates in group3, two replicates in group 4, and two replicates in group5. In order to test the interaction effect of the different disease, the meaningful contrast was considered to be group1—group2—group3 –group4 + control. Then, differentially expressed genes with P-value < 0.01 were selected as potential candidates in different cancer types.

### Cluster Analysis based on Hierarchical Clustering

In this study, two-way Hierarchical Clustering, based on correlation distance and average linkage, was used for gene expression data analysis. In two-way hierarchical clustering, related experiments and genes with similar expression patterns were combined together and were connected by a series of branches to identify which genes are the most important for experiment clustering. These branches are added to the left side and the top of the heat map.

### Visualization based on Heat Map

Graphical representation of the changes in genes behavior in cancer data can be represented by heat map. We used heat map to visualized expression level of 8 significantly deregulated genes via color codes. The significantly deregulated genes are shown in rows and the samples are shown in columns. Red, black, and green represent the levels of expression: downregulated, not changed, and upregulated, respectively.

### Target Analysis and miRNA Target Prediction

The potential targets of miR-145 were used as input and after doing statistical analysis, the selected putative target genes with P-value smaller than 0.01 was considered as statistically significant genes. STRING (http://string-db.org/) is a gene function prediction method that was used in this study for displaying the interactive functional association network and illustrating the relationships among the genes and data sets [23]. By using STRING we found the Protein-Protein Interaction Networks (PPI) of common miR-145 targets. In addition, pathway commons tool was used to analyze pathways where MYL9, UNG, TAGLN, FUCA2, DERA, GMFB, TF, and SNX24 were involved and to find out possible interacters (http://www.pathwaycommons.org/) [24].

Potential targets of mir-145 were predicted using the following in silico tools: miRwalk (http://zmf.umm.uni-heidelberg.de/apps/zmf/mirwalk2/) [25], TargetScan (http://www.targetscan.org) [26], miRBase http://microrna.sanger.ac.uk [27], miRANDA (http://www.microrna.org) [28], and miRDB (http://mirdb.org/miRDB/) [29].

## Results

The aim of this study was to identify common target genes of tumor suppressor miR-145 via meta-analysis of microarray-based gene expression profiles in several human cancer types. In this study, we collected a total of 7 gene expression profile data set from previously published studies considering the inclusion criteria. We retrieved the GSE Series Matrix files, platform sets and annotations files from the Gene Expression Omnibus (GEO) including 16 array samples using GEOquery package in Bioconducter with R version. Then, did transformation on raw data, found common genes, and removed batch effects.

Details of the each individual microarray studies are summarized in Table 1.

**Table 1:** A summary of the each individual microarray datasets from different GEO dataset.

Study Set	GEO Accession	The platform of dataset	Platform	Samples Containing Mir-145	References	Cancer Type
**1**	GSE47657	GPL13607	Agilent-028004 SurePrint G3 Human GE 8x60K Microarray	GSM1154161(PC3), GSM1154163(DU145), GSM1154165 (LNCap)	[38]	Prostate cancer
**2**	GSE24782	GPL10332	Agilent-026652 Whole Human Genome Microarray 4x44K v2	GSM610397(PC3), GSM610398(DU145)	[39]	Prostate cancer
**3**	GSE58295	GPL4133	Agilent-014850 Whole Human Genome Microarray 4x44K G4112F	GSM1406126 (PC3-8h), GSM1406127 (PC3-16h), GSM1406128 (PC3-24h)	[40]	Prostate cancer
**4**	GSE37119	GPL10332	Agilent-026652 Whole Human Genome Microarray 4x44K v2	GSM911053 (HNSCC, IMC-3)	[41]	Head and neck squamous cell carcinoma
**5**	GSE18625	GPL570	[HG-U133_Plus_2] Affymetrix Human Genome U133 Plus 2.0 Array	GSM462902, GSM462903, GSM462904, GSM462905	[42]	Colon cancer (Exclude)
**6**	GSE19737	GPL570	[HG-U133_Plus_2] Affymetrix Human Genome U133 Plus 2.0 Array	GSM492843, GSM492844, GSM492845	[43]	Breast cancer
**7**	GSE20028	GPL4133	Agilent-014850 Whole Human Genome Microarray 4x44K G4112F	GSM500946 (TE2), GSM500948 (TE13)	[44]	Esophageal squamous cell carcinoma
**8**	GSE19717	GPL4133	Agilent-014850 Whole Human Genome Microarray 4x44K G4112F	GSM492573 (KK47), GSM492575 (T24)	[44]	Bladder cancer

Data set 1 (GSE47657) is obtained from a microarray analysis, which was performed in human prostate cancer cell lines including PC3, DU145, and LNCaP cells, treated with miR-145 to investigate the differentially expressed genes using SurePrint G3 Human GE 8×60K Microarray (Agilent Technologies, Santa Clara, CA, USA) containing 62,976 probes.

In the data set 2 (GSE24782), The microarray analysis was generated from PC3 and DU145 human prostate cancer cell lines which were transfected with miR-145 were using Agilent-026652 Whole Human Genome Microarray 4x44K v2 with 44,495 probes.

Data set 3 (GSE58295) was generated from mir-145 transfected PC3 cells which were collected at 8, 16 and 24 hours after transfection along with untransfected control PC3 cells using Agilent-014850 Whole Human Genome Microarray 4x44K G4112F containing 45,015 probes.

Besides, data set 4 (GSE37119) is obtained from a microarray analysis which was performed in human head and neck squamous cell carcinoma cell lines HNSCC and IMC3 transfected with miRNA 145 utilizing Agilent-026652 Whole Human Genome Microarray 4x44K v2 to arrays spotted with 44,495 probes.

Data set 5 (GSE18625) microarray analysis was performed using DLD-1, colon carcinoma cell line, transfected with miR-145 and collected 24 hours after transfection. Gene expressions were profiled on Affymetrix Human Genome U133 Plus 2.0 Array containing 54675 probes.

Data set 6 (GSE19737) was generated from miR-145 or a negative control pre-miRNA transfected MDA-MB-231 cell line which is most typical cell line with highly metastatic features in breast cancer using Affymetrix Human Genome U133 Plus 2.0 Array with 54,675 probes.

Data set 7 (GSE20028) identified miR-145 targets in squamous cell carcinoma. The aim of study was to explore of miR-145 target genes using Agilent-014850 Whole Human Genome Microarray 4x44K G4112F which includes 45,015 probes.

Lastly, data set 8 (GSE19717) gene expression profiles of bladder cancer cell line KK-47, and urinary bladder cancer cell line T24 were investigated upon miR-145 transfection using Agilent-014850 Whole Human Genome Microarray 4x44K G4112F with 45,015 probes.

**Fig 3:**
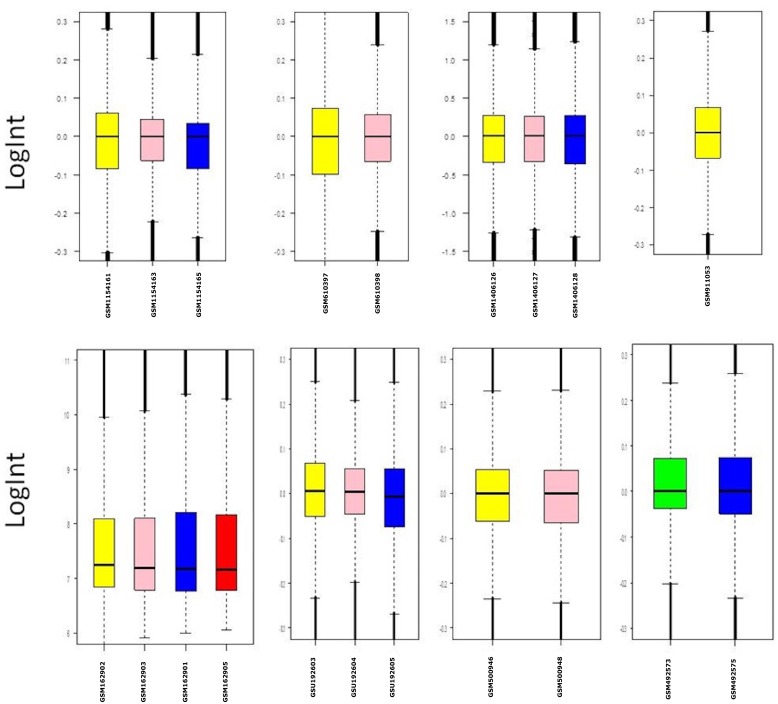
Box-plot representations of the GSM datasets.

**Fig 4:**
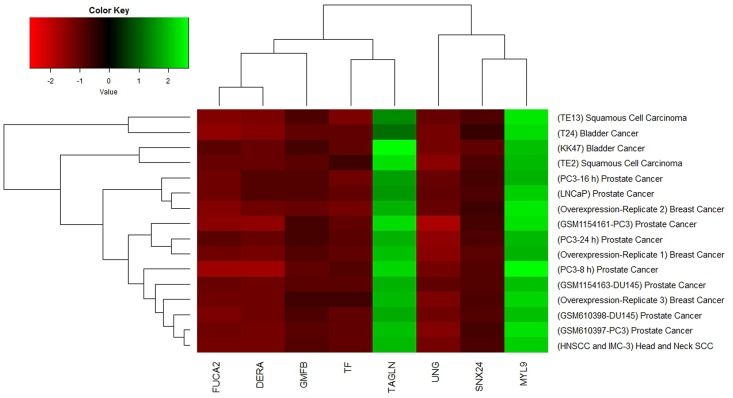
Heat map representation. Heat map representation of commonly deregulated genes by mir-145 overexpression in 5 types of cancer.

**Fig 5:**
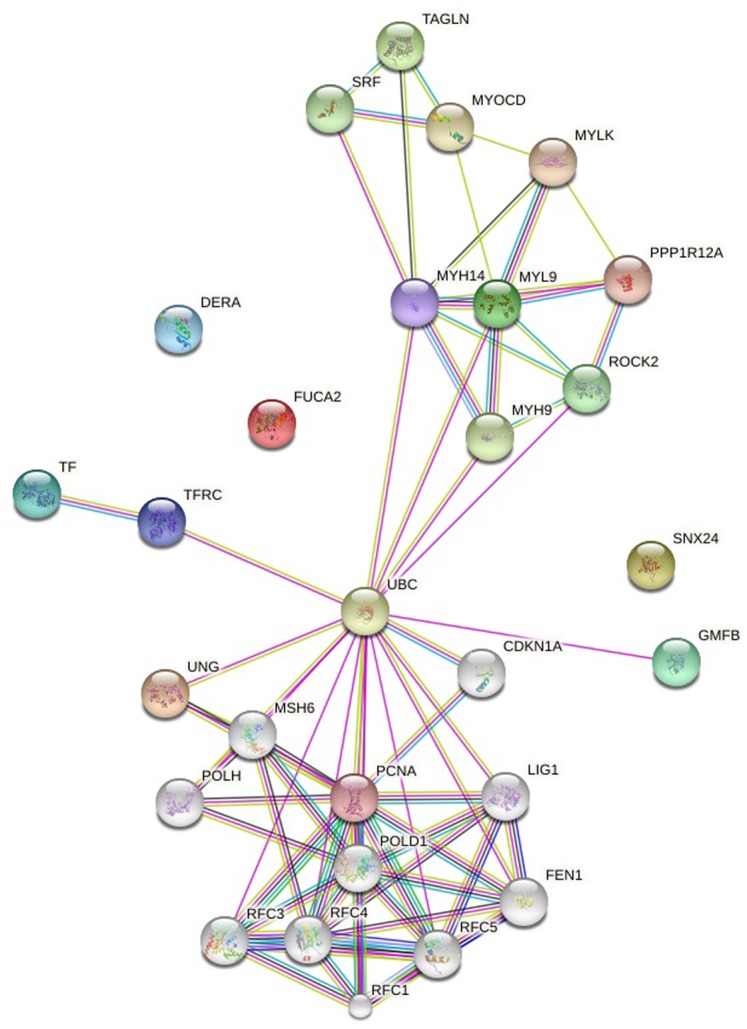
PPI network of commonly deregulated mir-145 targets. Pink: experimentally determined (known interactions), Blue: from curated databases (known interactions). Yellow: textmining, Green: gene neighborhood (Predicted interactions), Black: co-expression. The interaction score was set to high confidence (0.700).

**Fig 6:**
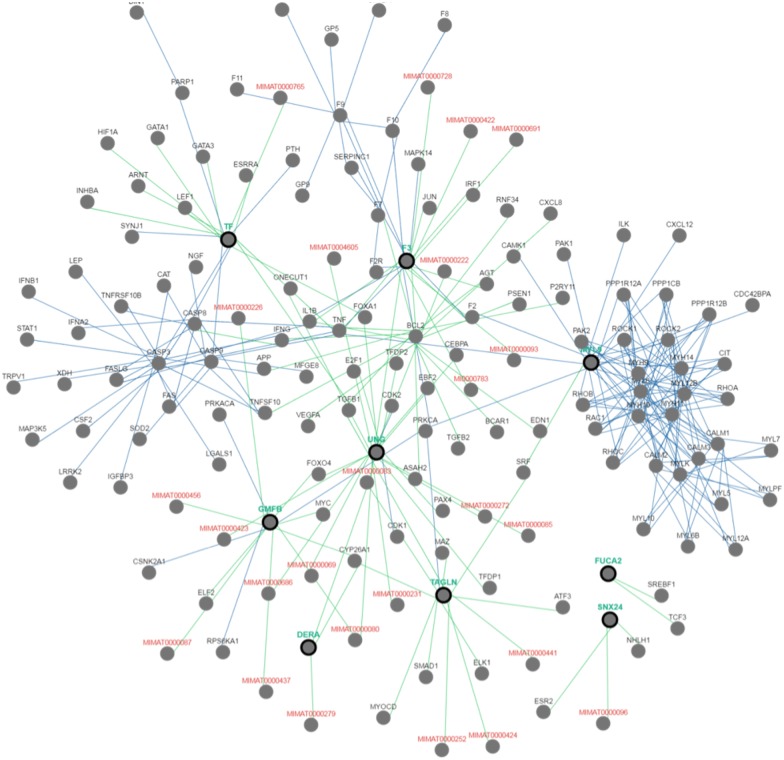
Pathway analysis of MYL9, UNG, TAGLN, FUCA2, DERA, GMFB, TF, and SNX24. Green: control expression, Blue: controls state change.

In order to clarify whether the microarray data were comparable, we initially prepared the Box plots representation of median-centered gene expression as provided in Fig 3. It shows the common target gene expression levels for probe set over all arrays. According to the result all GSE datasets were centered on zero except from GSE18625 (colon cancer). Therefore, we excluded this data set before the statistical analyses. Expression data in all GSEs were converted from (log_10_) to (log_2_) to eliminate variability among the datasets and batch effect was removed. Then, by making proper contras matrix significantly differentially expressed genes were found. As a result of the meta-analysis, we found that UNG, FUCA2, DERA, GMFB, TF, and SNX24 are significantly downregulated, and MYL9 and TAGLN are significantly upregulated in all GSM data. As a result, we found eight common target genes of mir-145 that have similar behavior in different GEO datasets. A heat map representation of these genes is demonstrated in Fig 4. In silico analysis tools predicted these genes as potential targets of miR-145 (Table 2). Biological process (Table 3), molecular function (Table 4), cellular component (Table 5), and KEGG pathways (Table 6) analysis of these potential targets of mir-145 through functional enrichments in PPI network, demonstrated that those genes are significantly involved in telomere maintenance, DNA binding and repair mechanisms. Besides, PPI network of commonly deregulated mir-145 targets and pathway analysis of MYL9, UNG, TAGLN, FUCA2, DERA, GMFB, TF, and SNX24 are represented in Figs 5 and 6, respectively.

**Table 2:** Representation of the potential targets of mir-145 by in-silico analysis.

Gene	EntrezID	RefseqID	miRWalk	miRanda	RNA22	Targetscan	SUM
UNG	7374	NM_003362	1	0	1	0	2
FUCA2	2519	NM_032020	0	1	0	1	2
DERA	51071	NM_015954	0	0	0	0	0
GMFB	2768	XM_005267541	1	0	1	1	3
TF	7018	NM_001063	0	0	1	0	1
SNX24	28966	NM_014035	0	1	0	1	2

**Table 3:** Biological process (GO) of the potential targets of mir-145 by functional enrichments in PPI network.

pathway ID	pathway description	count in network	false discovery rate
GO:0032201	telomere maintenance via semi-conservative replication	8	1.14e-14
GO:0000722	telomere maintenance via recombination	8	1.79e-14
GO:0006284	base-excision repair	9	1.79e-14
GO:0033260	nuclear DNA replication	8	1.79e-14
GO:0006271	DNA strand elongation involved in DNA replication	8	8.3e-14

**Table 4:** Molecular function (GO) of the potential targets of mir-145 by functional enrichments in PPI network.

pathway ID	pathway description	count in network	false discovery rate
GO:0003684	damaged DNA binding	5	5.91e-05
GO:0015091	ferric iron transmembrane transporter activity	2	0.00248
GO:0003689	DNA clamp loader activity	2	0.00446
GO:0003676	nucleic acid binding	15	0.00878
GO:0042623	ATPase activity, coupled	5	0.0109

**Table 5:** Cellular component (GO) of the potential targets of mir-145 by functional enrichments in PPI network.

pathway ID	pathway description	count in network	false discovery rate
GO:0005663	DNA replication factor C complex	5	5.39e-12
GO:0005657	replication fork	6	3.13e-08
GO:0044427	chromosomal part	9	4.64e-05
GO:0001725	stress fiber	4	0.000123
GO:0005654	nucleoplasm	15	0.000123

**Table 6:** KEGG Pathways of the potential targets of mir-145 by functional enrichments in PPI network.

pathway ID	pathway description	count in network	false discovery rate
03430	Mismatch repair	8	5.68e-16
03030	DNA replication	8	1.35e-14
03420	Nucleotide excision repair	7	1.97e-11
03410	Base excision repair	5	5.46e-08
04810	Regulation of actin cytoskeleton	6	1.84e-05

## Discussion

miRNAs are frequently located in the cancer-associated genomic regions or in fragile sites of the genome. In addition to in vitro and in vivo tools, bioinformatics approaches are of paramount importance to evaluate their roles in the pathogenesis of different types of cancer [30].

Expression of miR-145 has been commonly identified as downregulated in several human cancer types. Several studies suggested that low levels of miR-145 might contribute to pathogenesis and progression of human tumors [31, 32]. miR-145 is a well characterized tumor suppressor in human malignancies which targets various oncogenes in cancer cells. Functional analyses of target genes, which are repressed by mir-145, are crucial to explain their roles in cancer development.

The aim of the present study is to investigate the commonly targeted genes by mir-145 and relevant pathways through evaluating publicly available microarray datasets. We selected miR-145 in our meta-analysis due to its well-known function as a tumor suppressor and since its presence has been reported in a variety of cancers including prostate, esophageal, head and neck, breast, bladder cancer, and squamous cell carcinoma [33, 34]. In this study, we extracted 7 gene expression microarray datasets from GEO database (1 of them in Affymetrix Array, others Agilent), which are generated from cell line samples.

We identified six genes including UNG, FUCA2, DERA, GMFB, TF, and SNX24 as significantly downregulated and two genes including MYL9 and TAGLN as significantly upregulated upon mir-145 over-expression in distinct cancer types.

The tumorigenic potentials of those genes have not been studied extensively until now. Considering our results, we suggest them as important contributors in tumorigenesis, which should be demonstrated in in vitro and in vivo studies. UNG, FUCA2, DERA, GMFB, TF, and SNX24 as significantly downregulated upon mir-145 over-expression, are expected to have elevated expressions in tumor samples considering low levels of miR-145 in several cancer types. Among those genes, elevated expression of UNG, an essential enzyme for post-replicative repair of uracil in DNA [35], has been found to be associated with pemetrexed-resistance and present in cell lines derived from pemetrexed-resistant histologic subtypes [36]. Besides, high GMFB expression was related to poor disease-free survival and overall survival in patients with SOC (serous ovarian cancer) [37].

In order to minimize false positives, we used target prediction tools, including miRwalk, TargetScan, RNA22, and miRanda. The significantly downregulated genes that we found as a result of our meta-analysis were predicted to be targeted by mir-145 in at least one in-silico tools out of four. Interestingly, biological process, molecular function, cellular component, and KEGG pathways analysis of these potential targets of mir-145 through functional enrichments in PPI network showed that UNG, FUCA2, DERA, GMFB, TF, and SNX24 are significantly involved in telomere maintenance, DNA binding and repair mechanisms.

As a conclusion, our results pointed out the importance of mir-145 and its targets and suggested that they contribute to carcinogenesis in distinct tumor types.

To the best of our knowledge, this is the first study retrieving gene expression data from several cancer types and investigating the common targets of mir-145 to help enlightening the roles of mir-145 in cancer pathogenesis.
